# Supplementary material for: ARID5B drives an inflammatory-to-destructive shift in pathologic fibroblast behavior
Source: bioRxiv. 2026 Aug 10:2026.08.04.742822. Preprint. [Version 1] doi: 10.64898/2026.08.04.742822 (PMC13640481; doi:10.64898/2026.08.04.742822)
Supplement: Supplementary file 1 [file media-1.pdf]

Supplemental information

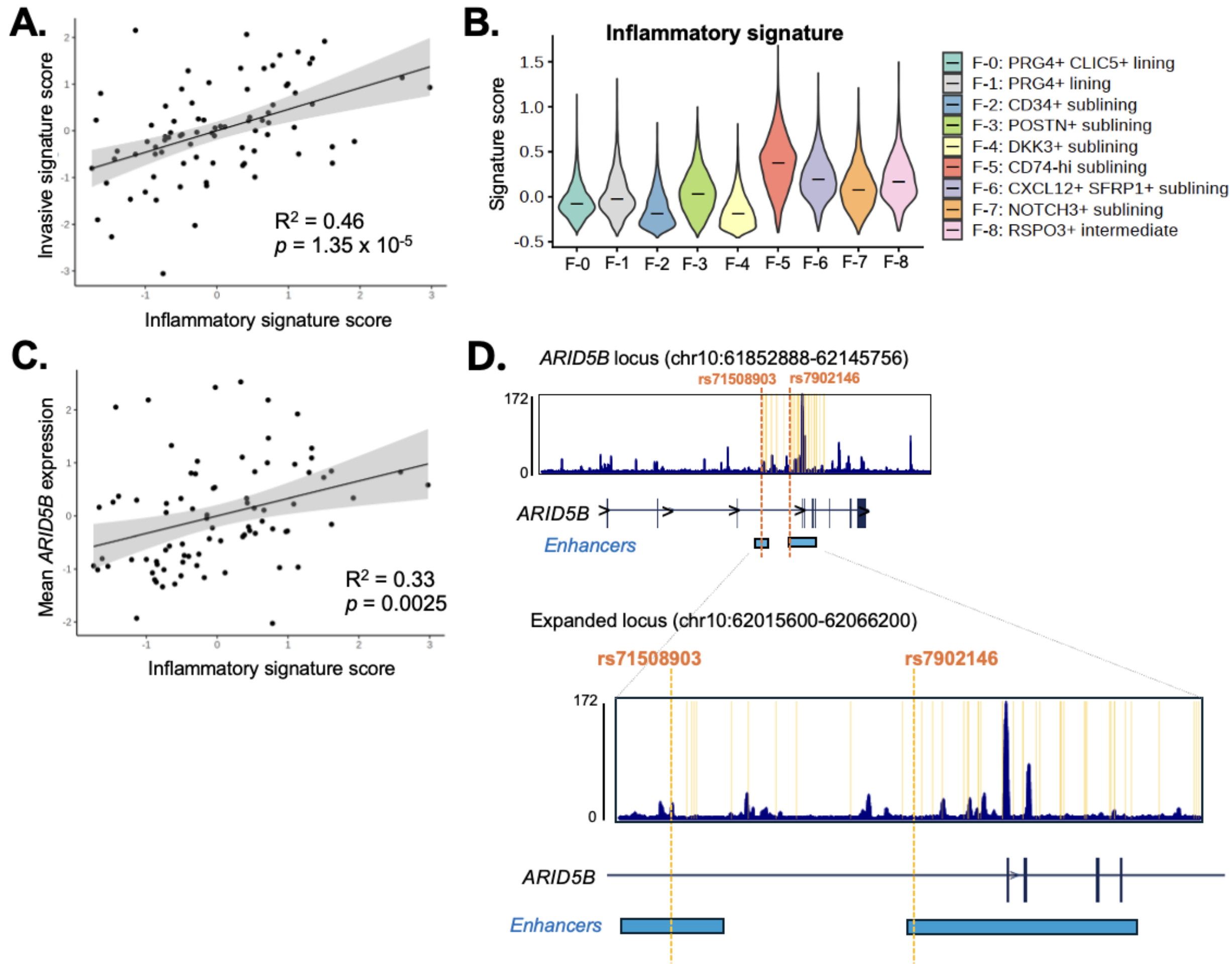

Figure S1. *ARID5B* expression across fibroblast populations and RA patients, related to Figure 1. (A) Scatterplot of scaled mean fibroblast inflammatory and invasive gene signature enrichment scores for each patient from the AMP RA/SLE Phase 2 study[9]. R^2 and p-value from Pearson correlation test. **(B)** Violin plots depicting inflammatory gene signature enrichment scores across the indicated synovial fibroblast subsets. Median expression indicated. **(C)** Scatterplot of scaled mean fibroblast inflammatory gene signature enrichment scores and *ARID5B* expression levels for each patient from the AMP RA/SLE Phase 2 study. R^2 and p-value from Pearson correlation test. **(D)** Track plot depicting ATAC-seq peaks along the *ARID5B* gene locus in synovial fibroblasts (averaged signals from n = 2 primary cell lines). Orange lines indicate genomic coordinates of SNPs significantly associated with RA risk ($p < 1 \times 10^{-8}$ in Ishigaki et al.[43]; lead SNPs labeled), shown in relation to the fibroblast ATAC-seq peaks and predicted *ARID5B* gene enhancer elements.

Table S1. Genes included in the inflammatory and invasive gene modules used in single-cell transcriptomic analysis of RA synovial fibroblasts, related to Figure 1.

Gene symbol	Category	Description	References (PMID)
IL6	Inflammatory	Pleiotropic cytokine with roles in T/B cell differentiation and activation and acute phase response, RA synovial fibroblasts are major source	32327746, 31061532
LIF	Inflammatory	IL-6 family member cytokine that amplifies RA synovial fibroblast inflammatory factor production via an autocrine signaling loop	1522240, 28228280
CXCL8	Inflammatory	Neutrophil chemoattractant and proangiogenic factor highly expressed by RA synovial fibroblasts	7561066, 11673556, 36725964
CXCL12 (SDF1)	Inflammatory	Monocyte and lymphocyte chemoattractant highly expressed by RA synovial fibroblasts	11086103, 11920421, 36725964
CCL2 (MCP1)	Inflammatory	Monocyte and lymphocyte chemoattractant, highly expressed by RA synovial fibroblasts and further stimulates their inflammatory factor production	33453247, 11673556
TNFSF13B (BAFF)	Inflammatory	B cell survival and maturation factor implicated in supporting autoreactive B cell activation and class-switching	21798884, 15634908, 19737141
HLA-DRA	Inflammatory	MHC class II molecule highly expressed by RA synovial fibroblasts, involved in antigen presentation to autoreactive T/B cells	28649674, 31061532
CD74	Inflammatory	Invariant chain; facilitates MHC class II molecule assembly and cell surface expression, highly expressed by RA synovial fibroblasts	37938773, 40093895
CCL19	Inflammatory	Chemokine for T cell recruitment and activation, highly expressed by RA synovial fibroblasts	21225692, 34651581
CCL20	Inflammatory	Chemokine for monocyte/T cell recruitment and activation, highly expressed by RA synovial fibroblasts	19447772, 24394994
CXCL1	Inflammatory	Neutrophil chemoattractant, highly expressed by RA synovial fibroblasts and further stimulates their inflammatory factor production	7561066, 33087182
CXCL3	Inflammatory	Chemokine for monocyte recruitment and angiogenesis, highly expressed by RA synovial fibroblasts	32079724, 36860872
CXCL6	Inflammatory	Chemokine for neutrophil recruitment, highly expressed by RA synovial fibroblasts	32079724, 20036936
VCAM1	Inflammatory	Binds leukocyte integrins to mediate leukocyte migration and tissue infiltration, highly expressed by RA synovial fibroblasts	8909250, 10820282
STAT1	Inflammatory	JAK/STAT transcription factor, significantly induced in cytokine-stimulated RA synovial fibroblasts	37938773, 14962955
CDH11	Invasive/Remodeling	Mesenchymal cadherin involved in synovial lining formation and RA synovial fibroblast-mediated joint destruction	17255475, 19404963, 22127696
MMP1	Invasive/Remodeling	Collagenase targeting intact fibrillar collagens, highly expressed by RA synovial fibroblasts	11714387, 37938773
MMP2	Invasive/Remodeling	Gelatinase degrading collagen II, highly expressed at the invasive pannus surface by synovial fibroblasts	9923652, 18471998
MMP3	Invasive/Remodeling	Aggrecanase; levels correlate with erosive damage in human RA, highly expressed by RA synovial fibroblasts	10556259, 37938773
MMP9	Invasive/Remodeling	Gelatinase degrading collagen monomers, enhances RA synovial fibroblast migration and invasion	14532148, 22960198
MMP13	Invasive/Remodeling	Collagenase targeting intact collagen II/fibrillar collagens and implicated in cartilage destruction, highly expressed by RA synovial fibroblasts	11714387, 18289056
MMP14	Invasive/Remodeling	Membrane-anchored proteolytic enzyme that activates other MMPs to facilitate ECM degradation, implicated in pannus invasion in RA synovium	9923652, 19248098
CD44	Invasive/Remodeling	Adhesion molecule that binds hyaluronan; highly expressed by RA synovial fibroblasts and modulates their migratory and invasive behavior	10943861, 15781582
PDPN	Invasive/Remodeling	Transmembrane glycoprotein promoting RA synovial fibroblast-mediated cartilage and bone destruction, possibly through invadopodia regulation	27863512, 25486435
TNFSF11 (RANKL)	Invasive/Remodeling	Cytokine required for osteoclastogenesis and bone erosion; RA synovial fibroblasts are major source	26025971, 16490750
EZR	Invasive/Remodeling	Crosslinker between actin cytoskeleton and plasma membrane molecules including CD44; promotes cell migration and invasion	24599913, 12711360
EGFR	Invasive/Remodeling	Growth factor receptor highly expressed on RA synovial fibroblasts, implicated in promoting invasiveness and osteoclast activation	31068444, 9683636, 22393153
HBEGF	Invasive/Remodeling	Growth factor highly expressed on RA synovial fibroblasts, implicated in promoting fibroblast motility and invasiveness	31068444, 9683636
PDGFRA	Invasive/Remodeling	Growth factor receptor highly expressed on RA synovial fibroblasts, implicated in promoting fibroblast proliferation, motility and invasiveness	1724096, 26704941
PDGFRB	Invasive/Remodeling	Growth factor receptor highly expressed on RA synovial fibroblasts, implicated in promoting fibroblast proliferation, motility and invasiveness	1724096, 26704941

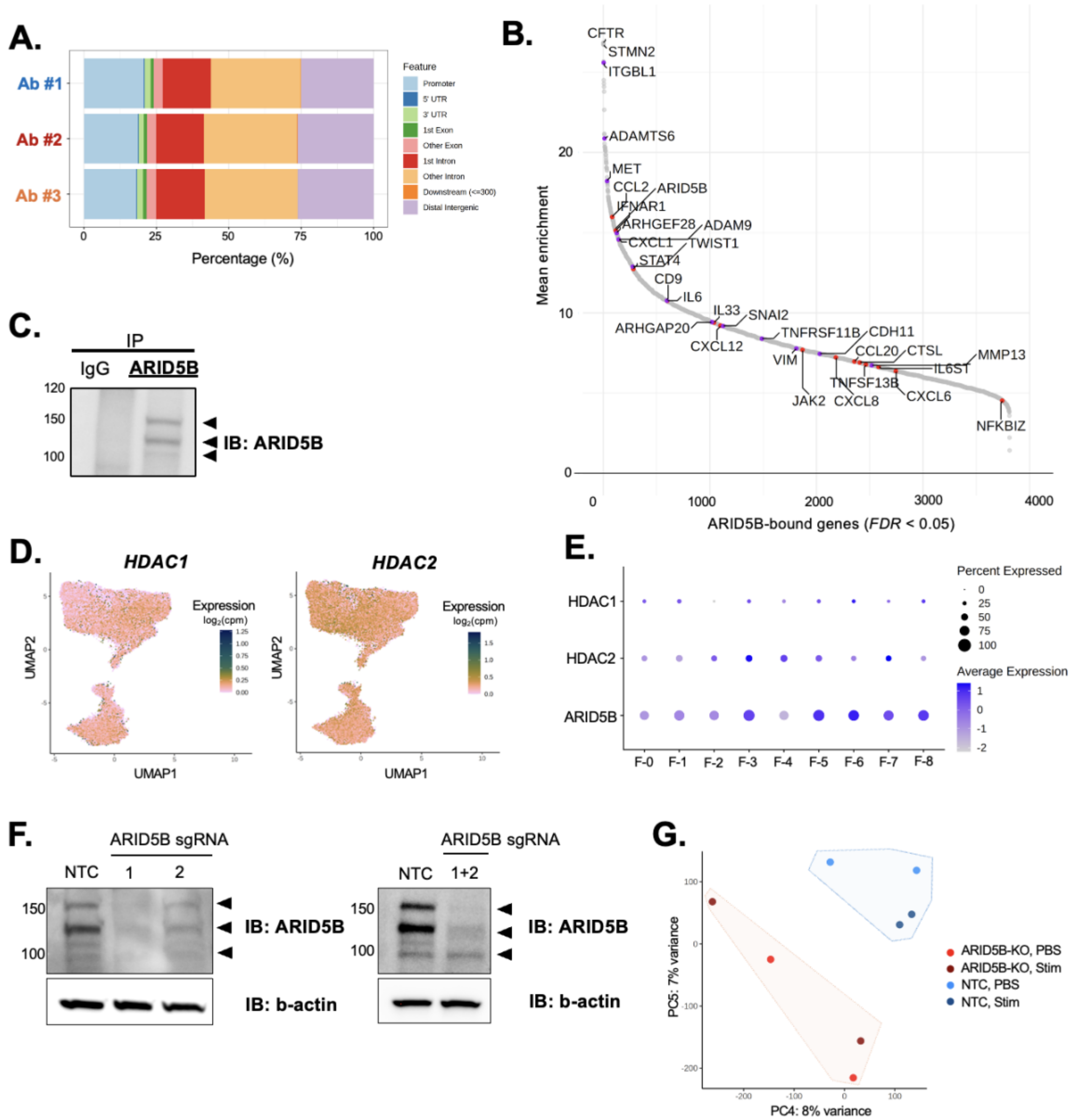

Figure S2. ARID5B genomic binding and epigenetic regulation, related to Figure 2. (A) Distribution of ARID5B binding sites in relation to annotated genomic features in synovial fibroblasts. Each row corresponds to a single CUT&RUN sequencing experiment performed using the indicated anti-ARID5B antibody. **(B)** Plot of mean peak enrichment values for genes with regulatory loci bound by ARID5B in all three ARID5B CUT&RUN sequencing reactions (*FDR* < 0.05). Purple points indicate genes associated with cell motility and invasion pathways; red points indicate genes associated with inflammation and immune regulation pathways. **(C)** Immunoblot for ARID5B following ARID5B immunoprecipitation from synovial fibroblast lysates; pulldown with nonspecific IgG shown as negative control. ARID5B isoforms indicated by arrows. **(D)** Projection of *HDAC1* and *HDAC2* expression levels across synovial fibroblasts in UMAP

space. **(E)** Dot plot of scaled mean and proportion of *HDAC1*, *HDAC2*, and *ARID5B* expression in each of the indicated synovial fibroblast subsets. **(F)** Immunoblot for ARID5B following CRISPR/Cas9-mediated deletion of ARID5B with the sgRNAs listed compared to wild-type non-targeting control sgRNA (NTC). ARID5B isoforms indicated by arrows. Beta-actin shown as cell lysate loading control. **(G)** PCA of normalized and scaled peak counts in wild-type (NTC) and ARID5B-deficient (KO) fibroblasts. Samples are colored by genotype and stimulation status.

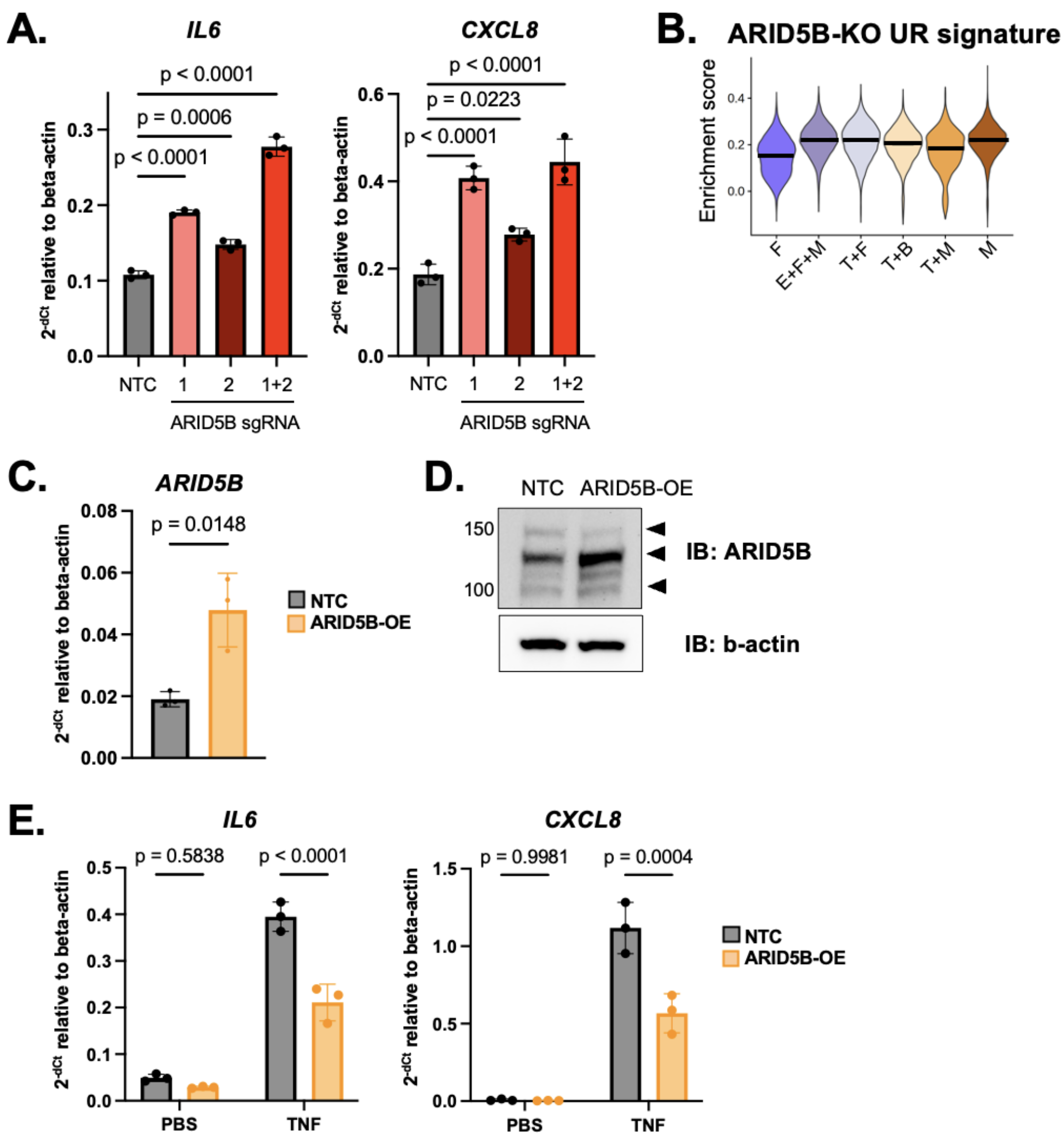

Figure S3. Inflammatory factor suppression by ARID5B, related to Figure 3. (A) RT-qPCR of *IL6* and *CXCL8* gene expression in TNFα-stimulated control (NTC) and ARID5B-deficient synovial fibroblasts. Mean ± SD shown. P values from one-way ANOVA with Dunnett multiple comparisons test. **(B)** Violin plot of ARID5B-upregulated gene signature scores across fibroblasts from all RA CTAPs. Median enrichment values shown. **(C)** RT-qPCR of *ARID5B* expression in fibroblasts following CRISPRa-mediated overexpression of *ARID5B* (OE) as compared to control (NTC). Mean ± SD shown. P value from two-sided Student's t test. **(D)** Immunoblot for ARID5B in ARID5B-overexpressing fibroblasts compared to control fibroblasts (NTC). ARID5B isoforms indicated by arrows. Beta-actin shown as cell lysate loading control. **(E)** RT-qPCR of *IL6* and *CXCL8* gene expression in unstimulated (PBS) and TNFα-stimulated control (NTC) and ARID5B-overexpressing (OE) synovial fibroblasts. Mean ± SD shown. P values from two-way ANOVA with Šídák's multiple comparisons test.

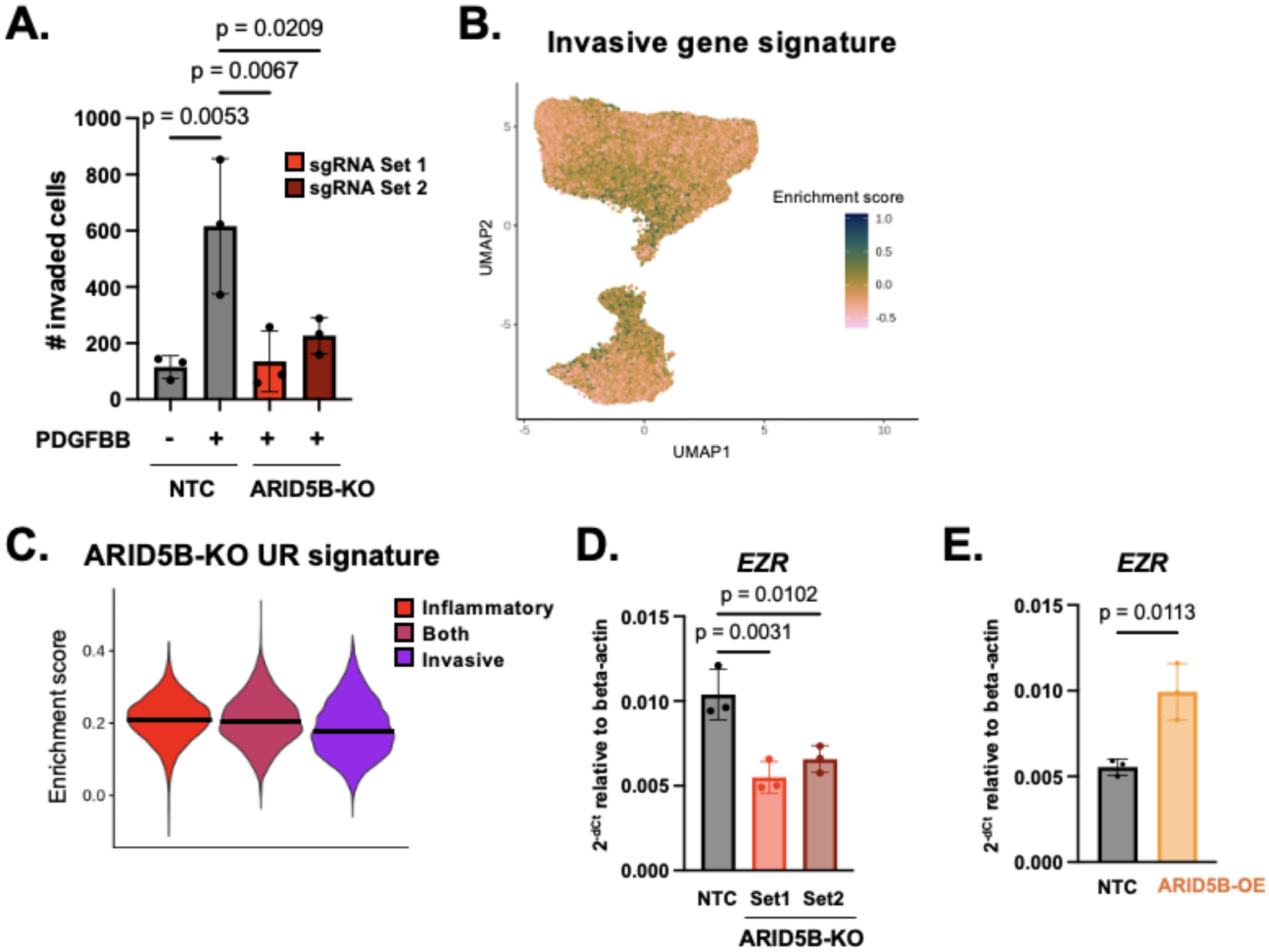

Figure S4. Upregulation of fibroblast invasion and *EZR* expression by ARID5B, related to Figure 4. (A) Enumeration of invaded cells by control (NTC) and ARID5B-deficient (KO) fibroblasts. Mean ± SD shown. P values from one-way ANOVA with Dunnett multiple comparisons test. **(B)** Projection of invasive gene signature enrichment scores across synovial fibroblasts in UMAP space **(C)** Violin plots of enrichment scores for the gene signature upregulated by ARID5B deletion in inflammatory versus invasive and mixed inflammatory-invasive synovial fibroblasts. **(D)** RT-qPCR of *EZR* gene expression in control (NTC) and ARID5B-deficient (KO) fibroblasts. Mean ± SD shown. P values from one-way ANOVA with Dunnett multiple comparisons test. **(E)** RT-qPCR of *EZR* gene expression in control (NTC) and ARID5B-overexpressing (OE) fibroblasts. Mean ± SD shown. P values from two-sided Student's t test.

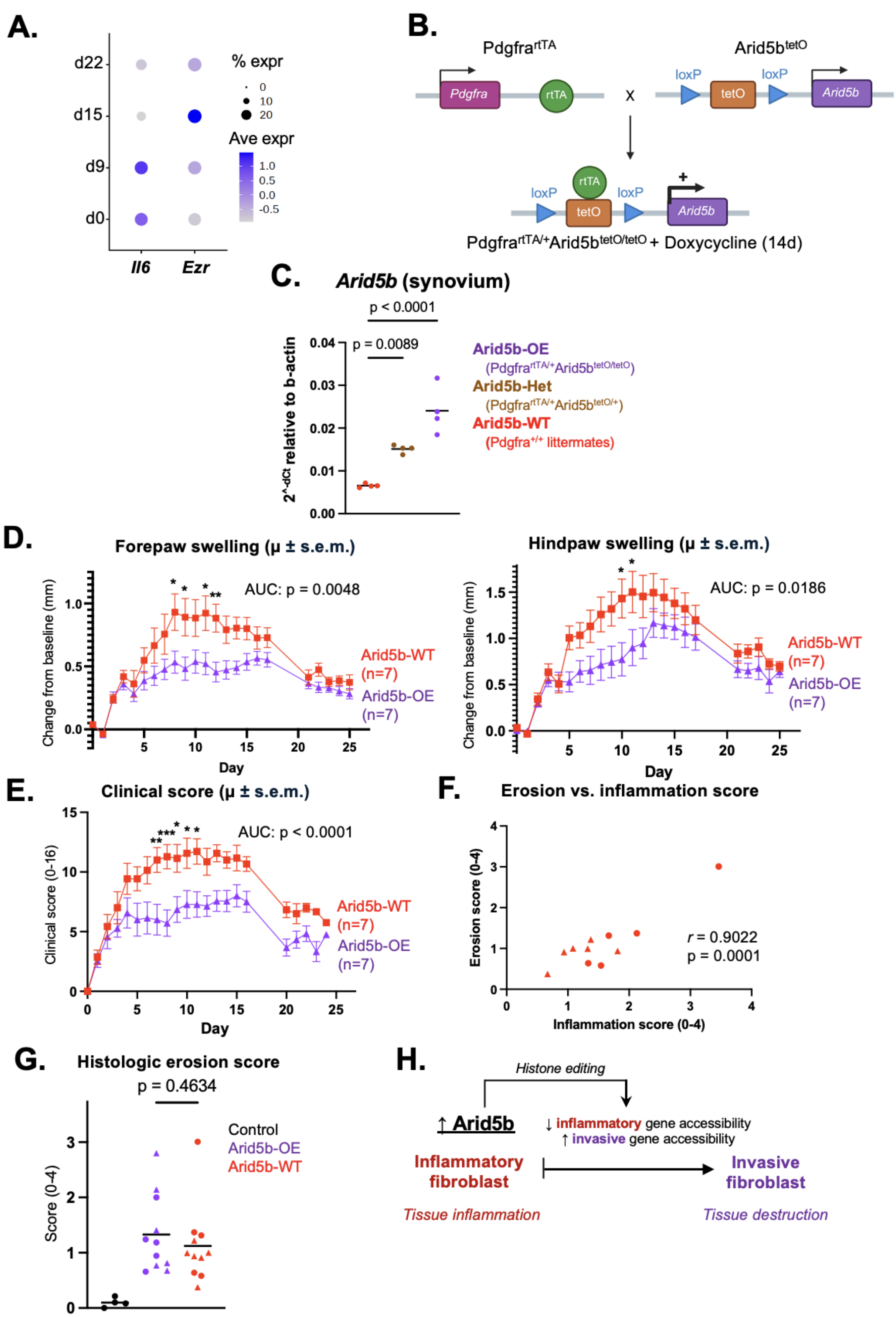
A.
d22
d15
d9
d0
Il6
Ezr
% expr
0
10
20
Ave expr
1.0
0.5
0.0
-0.5
B.
Pdgfra^{rtTA}
Arid5b^{tetO}
Pdgfra
rtTA
x
loxP
tetO
loxP
Arid5b
+
Pdgfra$^{rtTA/+}$Arid5b$^{tetO/tetO}$ + Doxycycline (14d)
C.
Arid5b (synovium)
p < 0.0001
p = 0.0089
2^-dCt relative to b-actin
Arid5b-OE
(Pdgfra$^{rtTA/+}$Arid5b$^{tetO/tetO}$)
Arid5b-Het
(Pdgfra$^{rtTA/+}$Arid5b$^{tetO/+}$)
Arid5b-WT
(Pdgfra$^{+/+}$ littermates)
D.
Forepaw swelling (μ ± s.e.m.)
AUC: p = 0.0048
Change from baseline (mm)
Day
Arid5b-WT (n=7)
Arid5b-OE (n=7)
Hindpaw swelling (μ ± s.e.m.)
AUC: p = 0.0186
Change from baseline (mm)
Day
Arid5b-WT (n=7)
Arid5b-OE (n=7)
E.
Clinical score (μ ± s.e.m.)
AUC: p < 0.0001
Clinical score (0-16)
Day
Arid5b-WT (n=7)
Arid5b-OE (n=7)
F.
Erosion vs. inflammation score
Erosion score (0-4)
Inflammation score (0-4)
r = 0.9022
p = 0.0001
G.
Histologic erosion score
p = 0.4634
Score (0-4)
Control
Arid5b-OE
Arid5b-WT
H.
Histone editing
↑ Arid5b
↓ inflammatory gene accessibility
↑ invasive gene accessibility
Inflammatory fibroblast
Invasive fibroblast
Tissue inflammation
Tissue destruction

Figure S5. Assessment of arthritic disease in mice with fibroblast-specific overexpression of Arid5b, related to Figure 5. (A) Dot plot of scaled mean and proportion of *Il6* and *Ezr* expression in synovial fibroblasts at each of the indicated STIA timepoints. **(B)** Schematic describing the generation of Arid5b-OE ($Pdgfra^{rtTA/+}Arid5b^{tetO/tetO}$) mice. **(D)** RT-qPCR of *Arid5b* gene expression in knee synovium harvested from Arid5b-OE, Arid5b-Het, and Arid5b-WT mice following two weeks of doxycycline administration. P values by one-way ANOVA. **(D-E)** Caliper measurements **(D)** and clinical scoring **(E)** of paw swelling and inflammation in Arid5b-OE mice (n = 7) and Arid5b-WT littermates (n = 7). Mean ± SEM shown at each timepoint. *p < 0.05; **p < 0.01; ***p < 0.001; ****p < 0.0001 by two-way repeated measures ANOVA with Šídák's multiple comparisons test. **(F)** Scatterplot of histologic erosion versus inflammation scores in Arid5b-WT mice (circles: n = 5 from STIA day 8; triangles: n = 6 from STIA day 14). *R* and p values by two-tailed Pearson's correlation test. **(G)** Histologic erosion scores in nonarthritic control (n = 4) and arthritic Arid5b-OE and Arid5b-WT mice (circles: n = 5 from STIA day 8; triangles: n = 6 from STIA day 14 per group). P value from two-sided Student's t test. **(H)** Model for Arid5b function in pathologic tissue fibroblasts.
